# Effect of brain-computer interface training based on non-invasive electroencephalography using motor imagery on functional recovery after stroke - a systematic review and meta-analysis

**DOI:** 10.1186/s12883-020-01960-5

**Published:** 2020-10-22

**Authors:** Antje Kruse, Zorica Suica, Jan Taeymans, Corina Schuster-Amft

**Affiliations:** 1 Department of Health Professions, Bern University Applied Science, Schwarztorstrasse 48, 3007 Bern, Switzerland; 2 Private Practice, Baslerstrasse 60, 4102 Binningen, Switzerland; 3 Research Department, Reha Rheinfelden, Salinenstrasse 98, 4310 Rheinfelden, Switzerland; 4 Vrije Universiteit Brussel, Pleinlaan 2, 1050 Brussels, Belgium; 5 Department of Engineering and Information Technology, Pestalozzistrasse 20, 3401 Burgdorf, Switzerland; 6 Department of Sport, Exercise and Health, University of Basel, Birsstrasse 320 B, 4052 Basel, Switzerland

**Keywords:** Stroke, Motor imagery, Brain computer interface training, Rehabilitation, Systematic review, Meta-analysis

## Abstract

**Background:**

Training with brain-computer interface (BCI) technology in the rehabilitation of patients after a stroke is rapidly developing. Numerous RCT investigated the effects of BCI training (BCIT) on recovery of motor and brain function in patients after stroke.

**Methods:**

A systematic literature search was performed in Medline, IEEE Xplore Digital Library, Cochrane library, and Embase in July 2018 and was repeated in March 2019. RCT or controlled clinical trials that included BCIT for improving motor and brain recovery in patients after a stroke were identified. Data were meta-analysed using the random-effects model. Standardized mean difference (SMD) with 95% confidence (95%CI) and 95% prediction interval (95%PI) were calculated. A meta-regression was performed to evaluate the effects of covariates on the pooled effect-size.

**Results:**

In total, 14 studies, including 362 patients after ischemic and hemorrhagic stroke (cortical, subcortical, 121 females; mean age 53.0+/− 5.8; mean time since stroke onset 15.7+/− 18.2 months) were included. Main motor recovery outcome measure used was the Fugl-Meyer Assessment. Quantitative analysis showed that a BCI training compared to conventional therapy alone in patients after stroke was effective with an SMD of 0.39 (95%CI: 0.17 to 0.62; 95%PI of 0.13 to 0.66) for motor function recovery of the upper extremity. An SMD of 0.41 (95%CI: − 0.29 to 1.12) for motor function recovery of the lower extremity was found. BCI training enhanced brain function recovery with an SMD of 1.11 (95%CI: 0.64 to 1.59; 95%PI ranging from 0.33 to 1.89). Covariates such as training duration, impairment level of the upper extremity, and the combination of both did not show significant effects on the overall pooled estimate.

**Conclusion:**

This meta-analysis showed evidence that BCI training added to conventional therapy may enhance motor functioning of the upper extremity and brain function recovery in patients after a stroke. We recommend a standardised evaluation of motor imagery ability of included patients and the assessment of brain function recovery should consider neuropsychological aspects (attention, concentration). Further influencing factors on motor recovery due to BCI technology might consider factors such as age, lesion type and location, quality of performance of motor imagery, or neuropsychological aspects.

**Trial Registration:**

PROSPERO registration: CRD42018105832.

**Supplementary information:**

**Supplementary information** accompanies this paper at 10.1186/s12883-020-01960-5.

## Background

The prevalence of stroke and the number of patients living with its consequences are increasing [1]. Advances in medical management of patients with stroke over the past decade have significantly reduced mortality, however, one third of the annually 16 million patients worldwide remain disabled [2]. Thus, more efficient stroke rehabilitation strategies are needed [1].

Brain-machine interfaces or brain-computer interfaces (BCI) in particular, are novel technologies enabling interaction with an individual’s environment through brain signals [3]. This technology records physiological measures of mental processes directly from the brain and decodes them into control signals that can operate external devices or a computer [4]. In recent years, such systems have been further developed to help patients after a stroke to regain their mobility and support motor function recovery by inducing activity-dependent brain plasticity [5]. Different types of input signals were used in development of clinical practice and research projects to control BCI. About 76.1% off all BCI Research Award submissions during the year 2015 used electroencephalography (EEG) based systems measuring motor imagery (MI) evoked potentials [5]. Other devices using non-invasive sensor systems are: Magnetoencephalography, functional Near-Infrared Spectroscopy and functional Magnetic Resonance Imaging. Invasive methods like subdural electrocorticography using action potential, intracortical local field potentials, and epidural field potentials [6] represent other possibilities for input signal sensors.

Motor imagery (MI) can be defined as the mental representation of movement without any overt body movement [6, 7]. This involves a visual or mental representation with or without a kinaesthetic feeling [6]. It is a complex cognitive operation, which is self-generated from the patients [8]. MI offers a unique opportunity for patients after a stroke, who are unable to move their extremities by attempting to stimulate the brain regions responsible for motor movement. MI combined with conventional therapy may improve outcomes more than conventional therapy only [9, 10]. BCI training (BCIT) systems can use EEG signals from MI performance with sensory real-time feedback and decode these signals to enable patients to direct devices such as personal computers, wheelchairs, robots, and prosthetic devices including exoskeletons. Some studies have investigated the efficacy of applying BCIT using MI on motor recovery for patients with subacute or chronic stroke with hemiparesis [3, 11–29]. Although these studies demonstrated a significant effect on recovery, the studies were flawed by low number of participants, low number of training sessions and/or a lack of follow-up assessments [3, 11–29].

We assumed that the efficacy of BCIT added to conventional therapy on motor function recovery of upper and lower extremity and brain function recovery is more beneficial than conventional therapy alone in patients after a stroke. The overall aim of our systematic review was to summarize the evidence from RCTs comparing BCIT to other therapy methods in patients after a stroke focusing on recovery of motor and brain function. The systematic review and meta-analyses aimed to answer the question: What is the effect of a brain-computer interfaces-based training with non-invasive EEG using MI on motor function and brain function recovery in patients after a stroke*?* We hypothesized that BCI training added to conventional therapy for motor function recovery of upper and lower extremity outperforms training without BCI technology. Furthermore, brain function recovery was evaluated as an objective parameter to indicate structural reorganisation in brain activity. Authors described different methods in their studies how they measured brain function recovery. Physiological measures that provide dynamic physiological information about brain function allow researchers to measure the contributions of various brain structures to specific psychological processes while participants complete motor or cognitive tasks. Functional brain measurement techniques [30] can measure increased regional blood flow, changes in oxygenation concentration during neural activities, glucose level, and brain cells communication by electrical impulses reflected by fluctuating lines in EEG recordings. Structural brain measurements [31] allow to examine the brain’s anatomical structure and to evaluate anatomical references, including tissue atrophy and white matter integrity. White matter integrity of premotor–motor connections is associated with motor output in chronic stroke patients [32].

## Methods

The review protocol was prepared according to the preferred reporting items for systematic review and meta-analysis protocols statements (PRISMA-P) [33] and was registered with the International Prospective Register of Systematic Reviews (PROSPERO, Registration number: CRD42018105832). The systematic review report was written following the Preferred Reporting Items for Systematic review and Meta-Analysis (PRISMA) guidelines and the PRISMA checklist [34].

### Search strategy, selection criteria and process

A systematic literature search was performed in Medline via EBSCO-Host surface, IEEE Xplore Digital Library, Cochrane library, and Embase. The US National Institute of Health’s ongoing trial register ‘clinical.trials.gov‘was searched to check for ongoing studies and unpublished reports. The search was carried out in July 2018 by a research librarian and was repeated for an update by the first author in March 2019. The search terms, strategy, and selection criteria are based on the PICOS system and were adapted for each database (see Additional file 1). Detailed inclusion criteria are listed in Table 1.

**Table 1:** Inclusion criteria based on PICOS-parameters

PICOS-Parameters	Inclusion criteria
**Population:**	Patients after a stroke (ischemic, hemorrhagic)
**Intervention:**	Brain-Computer Interface, Brain-Machine Interface
**Compare:**	Usual/conventional therapies
**Outcome:**	Assessments quantify motor function recovery in upper/ lower extremities or/and brain function recovery
**Study design:**	Controlled trials, randomised/randomized controlled trials

Our literature search was not restricted to any languages. All abstracts were available in English. None of the selected abstracts or full texts were excluded due to language. Studies were also excluded if they described interventions with animals or when full texts from authors were not available or were not formally peer reviewed. To decrease the risk of missing relevant studies, reference lists in the included studies were screened. Two independent reviewers (AK, ZS) screened for titles, abstracts and citations after removing duplicates from the eligible studies. To examine the agreement between the two reviewers (AK, ZS) in the pre-testing phase, 10 % of all studies were randomly selected and screened by both reviewers to check for congruence in selection. After screening of the titles and abstracts, full texts were evaluated. In case of disagreement, a third reviewer was consulted (CS) to decide on inclusion or exclusion of the study. Cohen’s Kappa statistic was used to evaluate the reviewer agreement [35].

### Data extraction

One researcher (AK) extracted the data from the selected studies. Data were manually entered into a Microsoft Excel (Version 14.0, 2010, Microsoft Corp., Redmond, California, USA) spreadsheet. The data extraction procedure was pilot tested on three studies, then reviewed, discussed and adjusted in accordance between the two reviewers (AK, ZS). After the pilot test, AK performed the complete data extraction and the second reviewer (ZS) crosschecked all extracted data. If needed, a consensus meeting and discussion resolved disagreement. A detailed description of the data collection process and a complete overview of extracted data are provided in Additional file 2. In case of incomplete data (e.g. only graphical presentations, missing *p*-values) in the selected studies, the corresponding authors were contacted to obtain the missing details.

### Assessment of risk of bias and GRADE

Two reviewers (AK, ZS) assessed risk of bias within studies using the Cochrane Collaboration risk of bias (RoB) 2.0 assessment [36]. Using the RoB 2.0 assessment six domains of bias were rated for every study, each domain having three rating categories: low RoB, moderate/some concerns RoB, and high RoB. One reviewer (AK) applied the RoB 2.0 assessment tool to judge the risk of over- or underestimating the effects of the intervention for the outcomes used for the meta-analysis. A second reviewer (ZS) crosschecked the completed RoB 2.0 assessment. Discussion between the reviewers (AK, ZS) resolved disagreement if needed.

The Grades of Recommendation, Assessment, Development and Evaluation (GRADE) was used to rate the overall quality of the evidence and the strength of the recommendations [37] . In accordance with the GRADE Working Group recommendations, the evidence was classified on four levels of quality: very low, low, moderate, and high quality. Moreover, publication bias was determined by computing a funnel plot.

### Data analysis

For all outcomes representing continuous data, means and standard deviations, sample size, and given *p*-values were entered into the Comprehensive Meta-Analysis Version 3.0 software (Biostat, Englewood, NJ, USA).

#### Primary outcomes

The weighted standardized mean differences (SMD) and their corresponding 95% confidence intervals (95%CI) were calculated for individual studies and visualised in forest plots separately for upper extremity, lower extremity, and brain function recovery. The analysis included the main outcome measure for motor function recovery or brain function recovery, as specified by the study investigator. The analysis for brain function recovery included the outcome measures such as indices of concentration plus changes in activation and connectivity in the brain function network. Different indices were used to analyse these responses of brain activity. However, if the study investigator did not specify the main outcome, the measure was specified by the first author (AK). Prior to the research, it was decided to meta-analyse the results of the individual studies using the random-effects model with the inversed-variance method due to expected heterogeneity between studies. To test the hypothesis of no among-studies heterogeneity the Q-test with its corresponding degrees of freedom (df) and *p*-value for an alpha level of 5% was used. Higgins’ I^2^ statistic was used as a measure of heterogeneity, indicating how much of the total observed variance can be explained by the true between studies variation [38]. T^2^ was used to measure the actual dispersion of variance [39]. In addition, the 95% prediction interval (95%CI) was calculated for the overall weighted mean estimate [39]. This indicator shows, in 95% of cases, that the true effect size in a new study will be within the range of the dispersion of the effect size [39]. The overall mean effect size was expressed in the original metric of the FMA scale. According to the Cochrane handbook in re-expressing SMDs using a familiar instrument, the standard deviation could be obtained as the pooled standard deviation of baseline scores [40].

#### Secondary outcomes

To evaluate effect size differences subgroup analyses were performed to compare the treatment effect for: (1) patients in the subacute (< 6 months after onset of stroke) and chronic (> 6 months after onset of stroke) period after stroke [39] (2) training intensity (high: 5 times per week vs. moderate: 2–3 times per week), (3) training duration (short: 2–3 weeks vs. long: 4–8 weeks, and (4) the follow-up period after training (short-term: 12 weeks vs. long-term: 24–36 weeks). Furthermore, a sensitivity analysis for RoB results was performed. Studies with overall bias ‘high’ in the RoB 2.0 Assessments were removed from the meta-analysis to determine the robustness of the pooled effect size [36]. To evaluate the impact of covariates in relation to the effect size, a meta-regression was performed using the random-effects model [39]. Level of impairment at baseline (FMA upper extremity), training duration in weeks (long, short), and the interaction between these two single covariates were chosen as moderators.

## Results

### Study selection

**Fig. 1:**
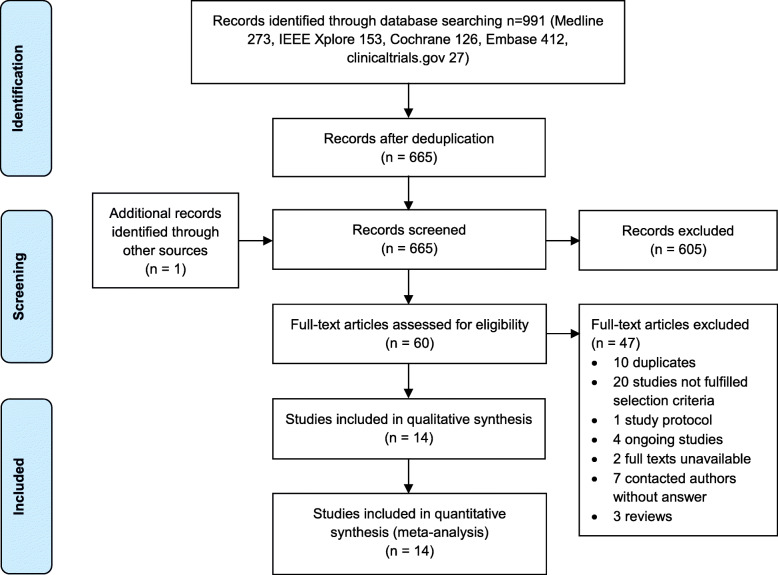
Reference selection process

In July 2018, the initial search was performed in the specified databases with the defined search strategy for the identification of studies. The identified 991 studies were imported into the reference management software Endnote (Clarivate Analytics, Philadelphia, USA). After reference import, 326 duplicates were removed, and 665 studies remained. No new references were identified by the search update in March 2019. Figure 1 illustrates the selection process.

Two reviewers (AK, ZS) independently examined whether the relevant studies fitted the population, intervention, comparison, outcome and study design (PICOS) strategy of our research question. After screening titles and abstracts of all studies, full texts of the remaining 61 studies were consulted. Eleven authors were contacted for missing data. Disagreement of selected full texts was resolved with mutual consent. Finally, fourteen studies could be included. The kappa statistic after full text screening was 0.82. The reviewers (AK, ZS) could not agree on three studies and therefore a third reviewer (CS) was consulted to decide on the studies` eligibility. The decision was to exclude the three studies. Table 2 provides an overview on all characteristics of the included studies.

**Table 2:** Characteristics of included studies

Authors	N BCI / Control	Age	Time after stroke	BCI intervention	Control Group	Outcome	Training duration	Training intensity	Baseline Fugl-Meyer UE (mean)
Ang, 2010 [16]	11/14	51.8	chronic	BCI-Manus robot	Manus robot	FM-UE	4 weeks	3 per week	14.9
Ang, 2014 [18]	6/8/7	54.2	chronic	BCI-Haptic knob	Haptic knob, standard arm therapy	FM-UE	4 weeks	3 per week	27.0
Ang, 2015 [14]	11/15	51.4	chronic	BCI-Manus robot	Manus robot	FM-UE, Brain symmetry index	4 weeks	3 per week	26.4
Biasuicci, 2018 [19]	14/13	58.0	subacute	BCI-FES	FES	FM-UE, Functional connectivity change	6 weeks	2 per week	21.6/19.9
Chung, 2015 [20]	5/5	43.6/50.2	chronic	BCI-FES	FES	Attention index Fp1 und Fp2 Activation index	5 days		–
Chung, 2015 [21]	5/5	43.6/50.2	chronic	BCI-FES	FES	TUG, BBS, cadence, affected sidestep lengths, affected side stride lengths	5 days		–
Curado, 2015 [22]	16/14	48.1	subacute	BCI-robotic orthosis	Sham	mFM-UE, EMG		5 per week	13.1/15.0
Frolov, 2017 [23]	55/19	58.0/58.0	subgroups: chronic and subacute	BCI-hand exoskeleton	Sham	FM-UE, ARAT, MAS	3 weeks	5 per week	19.5
Jang, 2015	10/10	61.1/61.7	subacute	BCI-FES	FES	MFT, shoulder subluxation x-ray classification, VAS	6 weeks	5 per week	–
Kim, 2016 [3]	15/15	59.5	chronic	BCI	Conventional therapy	FM-UE, MAL, mod. Barthel Index, RoM wrist flexion	4 weeks	5 per week	26.8/21.9
Mrachacz-K., 2015 [25]	13/9	46.3/50.2	chronic	BCI-EEG during foot dorsiflexion	Non assistive BCI-EEG	FM-LE, 10MWT, foot tapping frequency, corticospinal tract integrity, mRS	3 days		–
Pichiorri, 2015 [26]	14/14	64.1/59.6	subacute	BCI-virtual hand feedback	MI	mFM-UE, MRC, NIHSS, effectiveness factor FM, Laterality index in motor and premotor cortices	4 weeks	3 per week	23.4/24.2
Ramos-Murguialday, 2013 [27]	16/16	49.3/50.3	chronic	BCI-arm orthosis	Sham	mFM-UE, GAS, MAL, fMRI laterality index	8 weeks	2–3 per week	11.2/13.3
Varkuti, 2013 [28]	6/3	40.9/50.7	subacute 4 chronic 5	BCI-Manus robot	Manus robot	FM-UE, Functional connectivity change from RS-fMRI	4 weeks	3 per week	35.0/47.0

*Note:* Patients’ statistics (mean age and Baseline Fugl-Meyer Assessment) are published differently for the BCI-group and control groups or for the whole participant population

*Legend: ARAT* Action Research Arm Test, *BBS* Berg Balance Scale, *FES* Functional electrical stimulation, *FM-LE* Fugl-Meyer Assessment for the lower extremity (34 points), *FM-UE* Fugl-Meyer Assessment for the upper extremity (66 points), *10MW*T 10 m walking test, *fMRI* Functional magnetic resonance imaging, *Fp1, Fp2* Attention Index and Activation Index frontopolar 1 and 2, *GAS* Goal Attainment Scale, *MAL* Motor Activity Log, *MAS* Modified Ashworth Scale, *mFM-UE* Modified Fugl-Meyer Assessment without reflexes (54 points), *MFT* Manual Function Test, *MI* Motor imagery, *MRC* Medical Research Council scale for muscle strengths, *mRS* Modified Rankin Scale, *NIHSS* National Institute Health Stroke Scale, *RoM* Range of motion, *TUG* Timed up and go Test, *VAS* Visual Analogue Scale

### Characteristics of studies

Included studies were published between 2010 and 2017 including small sample sizes ranging between five to 55 patients (mean age between 40.9 and 64.1 years) in the subacute or chronic phase after a stroke in the experimental groups. The BCI training lasted for a minimum of 3 days to maximal 6 weeks with two to three training sessions per week. The Fugl-Meyer Assessment (FMA) for upper extremity was the most used assessment in ten studies, though three studies used a modified upper limb FMA with a maximal score of 54 points (not modified FMA max. Score = 226).

### Brain recovery indices

Four brain recovery indices were described. However not all indices could be included in the meta-analysis due to missing data:

1. Pichiorri et al. [26] and Ramos-Murguialday et al. [27] used the **laterality index** measured by fMRI to assess cerebral cortical lateralization to quantify brain recovery. In accordance to Pichiorri [26], the lateralization index expresses the normalized difference between the number of active voxels in the ipsilesional and contralesional. The lateralization index yields a value of 1 or − 1 when the activity was purely contralesional or ipsilesional respectively. Only patients with subcortical lesions were considered for a lateralization index assessment.
2. Várkuti et al. [28] and Biasiucci et al. [19] associated motor function recovery in patients in the chronic phase after stroke with quantitative indicators of functional neuroplasticity. The authors measured changes in functional connectivity after BCI by comparing the resting state fMRI pre- and post BCI training. According to the authors, the **functional connectivity changes** might reflect re-organisational processes that have occurred between pre- and post BCI training [28].
3. Chung et al. [14, 20] examined the effect of BCI based on functional electrical stimulation (FES) on **brain activation** in patients in the chronic phase after stroke. EEG brainwave patterns were calculated as the ratio of sensorimotor (SMR) and Mid-Beta waves to Theta waves ((SMR+ Mid-Beta)/Theta). The authors demonstrated significant effects for the EEG brainwave patterns in the frontopolar regions attention index 1 (Fp1) and 2 (Fp2), and frontopolar 1 (Fp1) [20].
4. A fourth index was mentioned by Ang et al. [14]. The authors collected EEG data during the BCI-Manus therapy to detect interhemispheric asymmetry using the **brain symmetry index** that can range between zero (lowest level of asymmetry) and one (highest level of asymmetry) [41]. The averaged brain symmetry index from all 12 sessions for the eleven participants in the BCIT group was analysed. Ang et al. and Anastasi et al. proposed that the brain symmetry index can be used as a prognostic measure for BCI-based stroke rehabilitation [14, 42]. However, the study from Ang et al. could not be included in the meta-analysis due to missing data from the control group.

### Risk of bias within studies

**Fig. 2:**
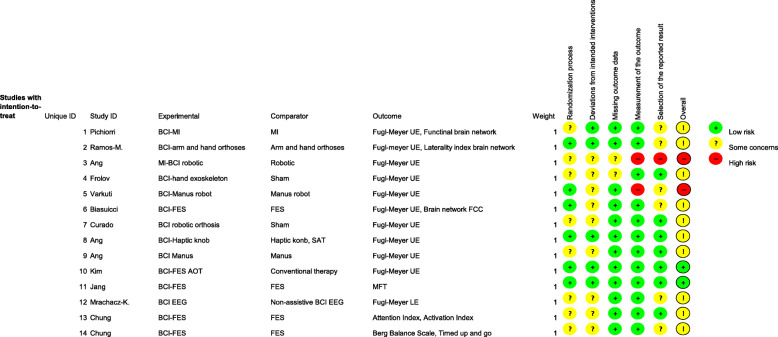
Risk of bias rating for each study. Legend: AOT = Action observation training, BCI=Brain computer interface, FES=Functional electrical stimulation, MFT = Muscle function test, MI = Motor imagery, UE = Upper extremity

**Fig. 3:**
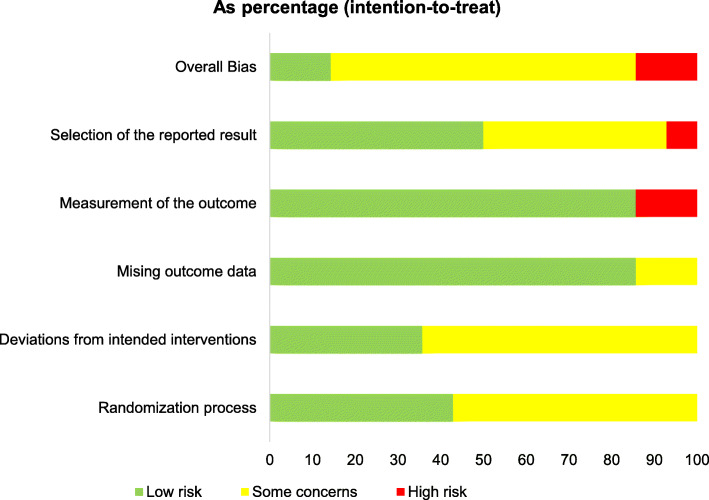
Domains rated as percentage of all studies

Figures 2 and 3 show the results of the RoB evaluation [36]. Qualitative assessment showed low risk in measurement of outcomes, missing outcome data, and selection of the reported results. Moreover, studies showed some concerns in the randomisation process and deviation from intended intervention. Only two studies had a high risk of overall bias.

### Risk of bias across studies

RoB results for all studies for the upper extremity were pooled and presented in funnel plots (Additional file 3). Looking for missing studies right of the mean, in accordance to the developed method, the CMA software adjusted two studies. Using the Trim and Fill method, the imputed point estimate was 0.43 (95%CI: 0.22 to 0.64). This indicates a slightly higher effect size, but still very similar to the point estimate of the pooled studies. The adjusted value was equivalent to the observed value and the weighted effect size was 0.39 (95%CI: 0.17 to 0.62). It can be concluded, if adjusting the effect to remove the possible bias or asymmetry, the resulting effect would remain unchanged. However, included studies did not indicate a significant asymmetry suggesting a low publication bias.

### GRADE- evidence profile table

After the evidence was summarised, small sample sizes, the width and overlap of confidence intervals, heterogeneity and generalisability were taken into consideration. One reviewer (AK) created a GRADE evidence profile table (Table 3) to present key information on five defined outcomes (GRADEproGDT, 2015). A second reviewer (ZS) crosschecked the results. The reviewers (AK, ZS) resolved disagreement by discussion. This was the case in the rating of the outcome ‘brain function recovery’ for ‘not serious’ or ‘serious’. The final decision was ‘serious’.

**Table 3:** Evidence profile table

Certainty assessment							Nr. of patients		Effect		Quality of evidence	Importance
Nr. of studies	Study design	Risk of bias	Inconsis-tency	Indirect-ness	Imprecision	Other considerations	BCI	Usual therapy	Relative(95% CI)	Absolute(95% CI)		
Motor function recovery upper extremity (follow-up: range 3 to 8 weeks; assessed with: Fugl-Meyer Assessment UE; scale from 0 to 54 or to 66 and MFT; scale from 0 to 32)												
11	randomised trials	not serious	not serious	not serious	not serious	none	174	155	–	SMD 0.39 SD (0.17 to 0.62)	⨁⨁⨁⨁HIGH	IMPORTANT
: Motor recovery after stroke lower extremity (follow-up: range 3 days to 5 days; assessed with: Fugl-Meyer LE, BBS; scale from: 0 to (FM) 34 or (BBS) 56)												
2	randomised trials	serious	not serious	serious ^a^	serious ^b^	none	18	14	–	SMD 0.41 SD (−0.29 to 1.12)	⨁◯◯◯VERY LOW	CRITICAL
: Brain function recovery (follow-up: range 5 days to 8 weeks; assessed with: Functional connectivity change indices, Attention index, Activation index and Lateralization index))												
5	randomised trials	not serious	not serious	not serious	serious	none	51	34	–	SMD 1.11 SD (0.64 to 1.59)	⨁⨁⨁◯MODERATE	IMPORTANT
: Short-term Follow-up (follow-up: mean 12 weeks; assessed with: Fugl-Meyer UE)												
4	randomised trials	serious ^c^	not serious	not serious	not serious	none	34	46	–	SMD 0.31 SD (−0.11 to −0.54)	⨁⨁⨁◯MODERATE	CRITICAL
: Long-term Follow-up (follow-up: range 24 weeks to 36 weeks; assessed with: Fugl-Meyer UE)												
2	randomised trials	not serious	not serious	not serious	serious ^d^	none	28	20	–	SMD 0.56 SD (0.01 to 1.11)	⨁⨁⨁◯MODERATE	CRITICAL

Legend: *Nr*. Number; *BCI* Brain-computer interface technology; usual therapy = without BCI; *CI* confidence interval; *FM-LE* Fugl-Meyer lower extremity; *FM-UE* Fugl-Meyer upper extremity; *MFT* Manual Function Test; *BBS* Berg Balance Scale; *SMD* standardised mean difference

a = too short intervention, no generalizability, no transferability; b = small sample size; c = reduced intensity for BCI group, 136 repetitions per session vs 1040 sessions in control group in one study and less motor imagery trials in BCI group in second study; d = wide CI (uncertain of magnitude of the effect)

### Primary outcomes- effect of BCIT on motor function recovery

**Fig. 4:**
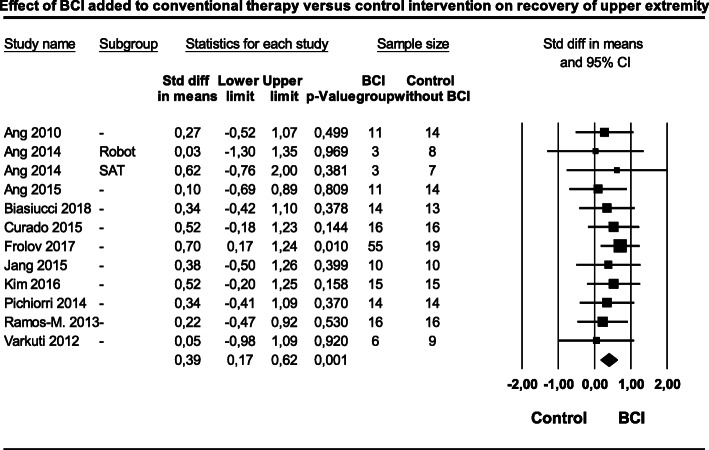
Effect of brain computer interface training added to conventional therapy versus control intervention on upper extremity motor function. Legend: BCI = Brain computer interface, CI=Confidence interval, SAT = Standard arm therapy, Std = Standard deviation

Eleven studies [3, 14, 16, 18, 19, 22–24, 26–28] with (329 patients in total) were included in a meta-analysis evaluating the effect of BCIT versus conventional therapy alone on motor function recovery of the upper extremity in patients after a stroke (Fig. 4). The weighted SMD was 0.39 (95% CI: 0.17 to 0.62) with a 95%PI ranging from 0.13 to 0.66 (Z = 3.45, *p* = 0.001). Heterogeneity was very low (I^2^ = 0.00; Q = 3.53, df = 11, *p* = 0.982). In this set of 11 studies the variance of the distribution of the effect sizes was T^2^ = 0.00.

**Fig. 5:**
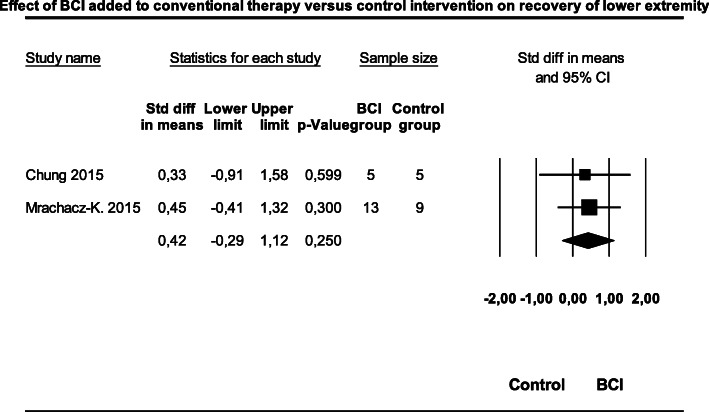
Effect of brain computer interface training added to conventional therapy versus control intervention on lower extremity function. Legend: BCI = Brain computer interface, CI=Confidence interval, Std = Standard deviation

Two studies [21, 25] were included in a meta-analysis for the effect of BCIT versus conventional therapy alone on motor function recovery of the lower extremity in patients after a stroke with 32 patients (Fig. 5). The weighted SMD was 0.41 (95%CI; − 0.29 to 1.12; Z = 1.15; *p* = 0.252). Heterogeneity was very low (I^2^ = 0.00; Q = 0.02; df = 1, *p* = 0.880). The variance of the distribution of the effect sizes in this sample of the two studies was T^2^ = 0.00.

**Fig. 6:**
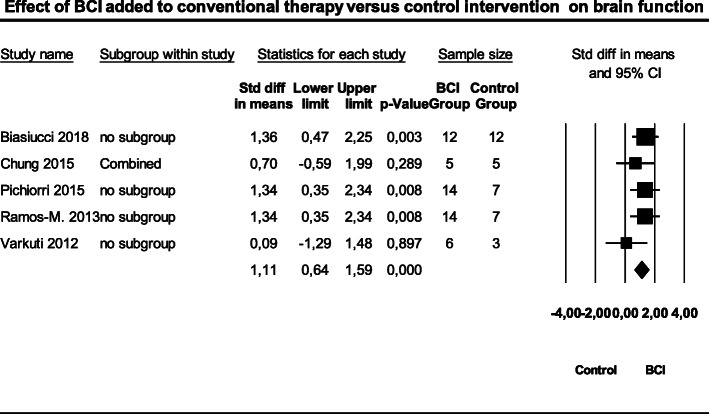
Effect of brain computer interface training added to conventional therapy versus control intervention on the brain recovery index. Legend: BCI = Brain computer interface, CI=Confidence interval, Std = Standard deviation

The meta-analysis, examining the effect of BCIT versus conventional therapy alone on brain function recovery in patients after a stroke, included five studies with 85 patients (Fig. 6) [19, 20, 26–28]. The overall weighted SMD was 1.11 (95% CI; 0.64 to 1.59) with a 95%PI ranging from 0.33 to 1.89 (Z = 4.82; *p* = 0.000). Heterogeneity was very low (I^2^ = 0.00; Q = 3.12, df = 5, *p* = 0.000). The variance of the distribution of the effect sizes in this sample of five studies was T^2^ = 0.00.

### Secondary outcomes

Different subgroups were defined to compare treatment effects and their influence on the effect size: different kinds of the BCIT, time since stroke onset, training intensity and duration, and follow-up period. The subgroup and sensitivity analyses regarding upper extremity motor function were performed and were presented in Additional files 4 and 5.

#### Subgroup analysis: time since stroke onset and training intensity

There is a statistically significant effect for both groups regarding time after stroke: subacute patients had a value of 0.57 (95%CI: 0.19 to 0.96; Z = 2.90; *p* = 0.004) and chronic patients a value of 0.39 (95%CI: 0.09 to 0.68; Z = 2.59; *p* = 0.010). Within the ‘moderate intensity’ subgroup, the weighted effect size was also in favour of the BCIT group as compared to the control group. However, the result was statistically not significant (*p* = 0.095). Within the ‘high intensity’ subgroup, the weighted effect size was 0.58 (95%CI: 0.24 to 0.92; Z = 3.33; *p* = 0.001) and statistically significant.

The weighted effect size for patients in the subacute or chronic phase after stroke can be interpreted as a small to medium effect.

#### Subgroup analysis: training duration and follow up period

Using the random-effects model, the weighted overall effect sizes were statistically significant for: (1) short duration training (two to 3 weeks) with a value of 0.54 (95%CI: 0.19 to 0.89; Z = 3.06; *p* = 0.002), (2) long duration training (four to 8 weeks) with a value of 0.31 (95%CI: 0.06 to 0.56; Z = 2.44; *p* = 0.016), and (3) long-term follow-up (24 to 36 weeks) with a value of 0.56. (95%CI: 0.01 to 1.11; Z = 1.99; *p* = 0.047).

The weighted effect size for short and long duration training, and long-term follow-up can be interpreted as a small to medium effect.

#### Sensitivity analysis

For the sensitivity analysis two studies [14, 27] were removed after being rated high-risk in the domain ‘overall bias’ (SMD = 0.42; 95%CI: 0.18 to 0.66; Z = 3.46; *p* = 0.001). The heterogeneity was very low (I^2^ = 0.00) and statistically not significant (Q = 2.97, df = 9, *p* = 0.965).

#### Meta-regression

A meta-regression was performed to assess the effect three covariates on the effect size: training duration, level of impairment, and the interaction between training duration and FMA baseline value. However, none of the three covariates showed a significant influence (see Additional file 6).

## Discussion

In this systematic review, 14 studies with patients after a stroke were included to investigate the effect of BCI on recovery after stroke. All studies focussed on the effect of BCIT added to conventional therapy on motor and brain function recovery in patients after a stroke ischemic or hemorrhagic stroke. Not all studies reported information regarding lesion location. However, if reported, the stroke was located in cortical and subcortical areas. Furthermore, studies involved more patients in the chronic phase after stroke than in the subacute phase after stroke. Most studies presented a low RoB in the outcome measurements, in the deviation from the intended intervention, and in the randomisation process. Studies focussing on motor function recovery for upper extremity were rated as ‘high quality’ and focussing on brain function recovery were rated as ‘moderate certainty’. Both study groups presented low sample sizes, wide CIs, and very short intervention periods. None of the included studies reported adverse events.

Meta-analyses indicated a statistically significant benefit of BCIT on motor function recovery of the upper extremity and brain function recovery in patients after a stroke. For the upper extremity, a statistically significant benefit could be interpreted as 5.4 to 8.1 points on the FMA of the upper extremity [3, 22, 23]. Furthermore, the analyses showed homogeneity among studies with the most participants and methodological sound experimental protocols. Only two trials focussed on motor function recovery of the lower extremity. However, both studies presented low sample sizes, large confidence intervals, and poor methodological quality making it impossible to draw clear-cut conclusions [21, 25].

Regarding the upper extremity, in a sensitivity analysis, two studies rated as ‘high risk’ in the RoB were excluded. The exclusion had a small effect on the overall weighted estimate indicating its robustness against RoB in the individual studies.

The subgroup analyses demonstrated significant effects for subacute and chronic patients. The weighted effect size for subacute patients had a large effect size of 0.57 compared to the small to medium effect size of 0.39 for chronic patients. Furthermore, for the high intensity training a large effect size of 0.58 was found whereas both the weighted effect size for short and long duration training was statistically significant. However, due to the trend to reach a recovery plateau in patients in the chronic phase after stroke, a high intensity BCIT (two to three times a week) with a training duration between three to 6 weeks might be an efficient approach in clinical practice.

To evaluate the impact of the covariates training duration, level of impairment, and the interaction between training duration and FMA baseline value on the effect size, a meta-regression was performed using the random-effects model [36]. However, no significant effects on the overall pooled estimate were detected. It remains to be investigated whether the training duration combined with the training intensity could have a significant influence on the effect size.

None of the 14 included studies used standardised assessments to evaluate MI ability. Malouin et al., described a mental slowing after stroke leading to more difficulties to generate a mental representation of movement [43, 44]. To determine participant benefits of BCIT based on MI, it is important to evaluate his/her MI ability [45]. Different valid and reliable assessments to evaluate patients MI ability are available such as the Kinaesthetic and Visual Imagery Questionnaire [46].

Furthermore, there were no standards for introduction or training of MI reported and there might be differences in the MI methods applied among facilities. A detailed description would be helpful to transfer the successfully implemented MI strategies into clinical routine. We further speculate that the potential of BCIT might be even higher if patients would receive a systematic MI introduction and training [47, 48].

A correlation between brain function recovery and motor function recovery was mentioned by Ramos-Murguialday et al. [27]. Authors mentioned a correlation between the laterality index changes and FMA subscale hand score (*r* = 0.54, *p* = 0.05). Pichiorri et al. examined, whether the possible changes in the intra-hemispheric networks correlated with the FMA score [26]. Their analysis detected a positive correlation between the increase in the laterality index value and the scoring in the FMA scale in the BCI group in the beta 1 bands (*r* = 0.57, *p* = 0.034), beta 2 (*r* = 0.60, *p* = 0.024), and gamma bands (*r* = 0.61, *p* = 0.023). The same laterality index was not significant for the unaffected hemisphere.

Moreover, Ang et al. [14] reported a correlation (*r* = − 0.62, *p* = 0.044) between the brain symmetry index, indicating a trend towards less asymmetry values and an improved FMA scoring.

Correlations between brain function recovery and cognitive assessments were not reported in the literature. However, the evaluation of cognitive capacities in patients with severe motor disabilities might have relevant implications for the BCIT systems [49]. Current BCIT systems are not well suited for use outside the clinic or research laboratory due to their large size, high costs, and lengthy set up time. Furthermore, the BCIT systems require highly trained personnel. However, future investigations should involve controlled experiments using low-cost BCIT systems. A section ‘state of the art’ of studies about costs and additional information about studies is provided in the supplementary material.

### Limitations and strengths

The systematic review process might have been confounded by some factors. The search was only performed in English language databases that published abstracts in English. However, the main literature sources are English-speaking peer-reviewed journals and conferences. No abstract or full text were excluded because it was published in a different language than English. The calculated effect sizes ignored the fact that conventional therapy compared with BCIT varied among the 14 included studies. However, the composition of the search strategy and the search itself were conducted by a professional research librarian from the University of Zurich in accordance with the review protocol providing a comprehensive search and detailed knowledge of different databases with a medical or technical focus.

## Conclusion

Based on the results of the present review, we recommend a BCIT combined with conventional therapy for a duration of 4 weeks or longer, with a preference for high intensity training of five times per week. BCIT combined with a wide range of different interventions reflects complexity and variety in of its applicability. We further recommend implementing assessments of neuropsychological parameters such as attention, concentration or cognition as well as MI ability measures, such as the KVIQ, to evaluate patients’ capabilities important for the mental training. An important topic for future work will be the examination of motor function recovery of the lower extremities in patients after a stroke. Furthermore, there is abundant scope for further progress in determining different aspects of the effect of BCIT on brain recovery in addition to functional connectivity. To develop a full picture of clinical factors that influence the effect size in motor recovery of upper extremities of patients after stroke, additional studies are needed to analyse parameters, such as patients’ age, lesion location, performance of MI ability, affected hemisphere, or type of lesion. Moreover, future studies should include long-term follow-ups 24–36 weeks after intervention begin in patients after a stroke. The interaction between baseline Fugl-Meyer Assessment score and training duration showed a no-significant effect. However, the interaction between the covariates training duration and training intensity should be further investigated.

## Supplementary information

**Additional file 1.** Example search strategy for each database.

**Additional file 2.** Overview on extracted study data.

**Additional file 3.** Figure AM1_Funnel plots.

**Additional file 4.** Figure AM2_Subgroup analyses: Time since stroke, training intensity and sensitivity analysis.

**Additional file 5.** Table AM1_Subgroup analysis: Time duration and FU.

**Additional file 6.** Figure AM3_Meta Regression

## Data Availability

All data presented in this systematic review are derived from published studies and are available from the first author on reasonable request.

## Abbreviations

BCI: Brain-computer interface
SMD: Standardized mean difference
CI: Confidence interval
PI: Prediction interval
EEG: Electroencephalography
MEG: Magnetoencephalography
fNIRS: Functional near-infrared spectroscopy
fMRI: Functional magnetic resonance imaging
ECoG: Electrocorticography
LEPs: Local field potentials
EFPs: Epidural field potentials
MI: Motor imagery
RoB: Risk of bias
GRADE: Grades of recommendation, assessment, development and evaluation
df: Degrees of freedom
FMA: Fugl-meyer assessment
KVIQ: The kinaesthetic and visual imagery questionnaire
