# Supplementary material for: Effect of brain-computer interface training based on non-invasive electroencephalography using motor imagery on functional recovery after stroke - a systematic review and meta-analysis
Source: BMC Neurol. 2020 Oct 22;20:385. doi: 10.1186/s12883-020-01960-5 (PMC7584076; doi:10.1186/s12883-020-01960-5)
Supplement: Supplementary file 1 — Additional file 1. Example search strategy for each database. [file 12883_2020_1960_MOESM1_ESM.pdf]

IEEE (112)

(("Document Title":"brain-computer interface" OR "Document Title":"brain-machine interface" OR "Document Title":"usercomputer interface" OR "Document Title":"human-computer interface" OR "Abstract":"brain-computer interface" OR "Abstract":"brain-machine interface" OR "Abstract":"user-computer interface" OR "Abstract":"human-computer interface") AND ("Document Title":stroke OR "Document Title":CVA OR "Document Title":"post-stroke" OR "Document Title":apoplex* OR "Abstract":stroke OR "Abstract":CVA OR "Abstract":"post-stroke" OR "Abstract":apoplex*))

Embase (174)

#6 #5 AND ([english]/lim OR [german]/lim)

#5 #4 NOT ([animals]/lim NOT [humans]/lim)

#4 #1 AND #2 AND #3

#3 'rehabilitation'/exp OR 'rehabilitation medicine'/exp OR 'daily life activity'/exp OR rehabilitation:ti,ab OR readaption:ti,ab OR readjust*:ti,ab OR revalidat*:ti,ab OR recover*:ti,ab OR 'functional assessment*':ti,ab OR neurorehabilitation:ti,ab OR 'neuro-rehabilitation':ti,ab OR independen*:ti,ab OR (((walking OR motor) NEAR/3 function):ti,ab) OR (((functional OR motor) NEAR/3 (recover* OR impair*)):ti,ab) OR ((daily NEAR/3 activit*):ti,ab)

#2 'cerebrovascular accident'/exp OR 'cerebrovascular disease'/exp OR 'brain ischemia'/de OR 'brain hemorrhage'/exp OR 'stroke patient'/exp OR stroke:ti,ab OR cva:ti,ab OR 'post stroke':ti,ab OR 'post-stroke':ti,ab OR apoplex*:ti,ab OR (((brain OR cerebral OR cerebrovascular OR ischaemic OR ischemic OR intracran* OR intracerebral OR subarachnoid) NEAR/3 (insult* OR attack* OR accident* OR arrest* OR injur* OR insufficien* OR failure* OR seizure* OR infarct* OR thrombo* OR emboli* OR occlus* OR haemorrhage* OR hemorrhage* OR haematoma* OR hematoma* OR bleed*)):ti,ab)

#1 'brain computer interface'/exp OR ((((brain OR human OR user) NEAR/1 (computer OR machine)):ti,ab) AND interfac*:ti,ab)

Cochrane (87)

#1 (((brain or human or user) near/1 (computer or machine)) and interfac*):ti,ab,kw (Word variations have been searched)

#2(stroke or CVA or post-stroke or "post-stroke" or apoplex*) or ((brain or cerebral or cerebrovascular or ischaemic or ischemic or intracran* or intracerebral or subarachnoid) near/3 (insult* or attack* or accident* or arrest* or injur* or insufficien* or failure* or seizure* or infarct* or thrombo* or emboli* or occlus* or haemorrhage* or hemorrhage* or haematoma* or hematoma* or bleed*)):ti,ab,kw (Word variations have been searched)

#3 (rehabilitation or readaption or readjust* or revalidat* or recover* or "functional assessment*" or neurorehabilitation or "neuro-rehabilitation" or independen*) or ((walking or motor) near/3 function) or ((functional or motor) near/3 (recover* or impair*)) or (daily near/3 activit*):ti,ab,kw (Word variations have been searched)

#4 #1 and #2 and #3

Medline (271)

S1 (MH "Brain-Computer Interfaces") OR TI (((brain OR human OR user) N1 (computer OR machine)) AND interfac*) OR AB (((brain OR human OR user) N1 (computer OR machine)) AND interfac*)

S2 ((MH "Stroke+") OR (MH "Cerebrovascular Disorders+") OR (MH "Brain Ischemia+") OR (MH "Intracranial Hemorrhages+")) OR TI ((stroke OR CVA OR post-stroke OR post stroke OR apoplex*) OR ((brain OR cerebral OR cerebrovascular OR ischaemic OR ischemic OR intracran* OR intracerebral OR subarachnoid) N3 (insult* OR attack* OR accident* OR arrest* OR injur* OR insufficien* OR failure* OR seizure* OR infarct* OR thrombo* OR emboli* OR occlus* OR haemorrhage* OR hemorrhage* OR haematoma* OR hematoma* OR bleed*))) OR AB ((stroke OR CVA OR post-stroke OR post stroke OR apoplex*) OR ((brain OR cerebral OR cerebrovascular OR ischaemic OR ischemic OR intracran* OR intracerebral OR subarachnoid) N3 (insult* OR attack* OR accident* OR arrest* OR injur* OR insufficien* OR failure* OR seizure* OR infarct* OR thrombo* OR emboli* OR occlus* OR haemorrhage* OR hemorrhage* OR haematoma* OR hematoma* OR bleed*)))

S3 ((MH "Rehabilitation+") OR (MH "Physical and Rehabilitation Medicine+") OR (MH "Activities of Daily Living")) OR TI ((rehabilitation OR readaption OR readjust* OR revalidat* OR recover* OR functional assessment* OR neurorehabilitation OR neuro-rehabilitation OR

independen*) OR ((walking OR motor) N3 function) OR ((functional OR motor) N3 (recover* OR impair*)) OR (daily N3 activit*)) OR AB ((rehabilitation OR readaption OR readjust* OR revalidat* OR recover* OR functional assessment* OR neurorehabilitation OR neuro-rehabilitation OR independen*) OR ((walking OR motor) N3 function) OR ((functional OR motor) N3 (recover* OR impair*)) OR (daily N3 activit*))

S4 S1 AND S2 AND S3

S5 S4 NOT (MH "Animals" NOT MH "Humans")

S6 S5 AND LA (English OR German)
