# Supplementary material for: Effect of brain-computer interface training based on non-invasive electroencephalography using motor imagery on functional recovery after stroke - a systematic review and meta-analysis
Source: BMC Neurol. 2020 Oct 22;20:385. doi: 10.1186/s12883-020-01960-5 (PMC7584076; doi:10.1186/s12883-020-01960-5)
Supplement: Supplementary file 2 — Additional file 2. Overview on extracted study data. [file 12883_2020_1960_MOESM2_ESM.pdf]

Overview on extracted study data.

Study-related data	1. Author<br>2. Title<br>3. Year of publication<br>4. Country of study
Population- and disease-related data	1. Diagnosis (ischemic, hemorrhagic stroke)<br>2. Affected body side<br>3. Affected brain location<br>4. Severity of stroke<br>5. Time after stroke<br>6. Age (mean), gender<br>7. Trial's inclusion and exclusion criteria
Methodological data	1. Study design<br>2. Randomization, by whom, how done, how concealed<br>3. Blinding, who, what blinding checked<br>4. Incomplete outcome reporting<br>5. Proportion of patient drop-outs<br>6. Followed the analysis of the intention-to-treat principle<br>7. Selective outcome reporting
Experimental group	1. Sample size<br>2. BCI screening<br>3. Drop-outs<br>4. Measurement sessions
BCI-training-related measures	1. BCI technology used<br>2. Duration<br>3. Frequency<br>4. Total number of trainings<br>5. Total number of training time<br>6. Training content<br>7. Training level recommendations and background for personnel
Comparison group	1. Sample size<br>2. Drop-outs<br>3. Measurement sessions
Comparison: training related measures	1. Type or technique used<br>2. Duration<br>3. Frequency<br>4. Total number of trainings

	5. Total number of training time<br>6. Training content<br>7. Training level recommendations and background for personnel
Outcomes	1. Pre- and post-assessment data (raw and/or data that report changes in the assessment from pre- to post-measurement session) regarding<br>a. Upper limb function<br>b. Lower limb function<br>c. Walking ability<br>d. Balance<br>e. Activity of daily living<br>f. Any other functional or impairment-related assessment<br>2. Raw or change in term of accuracy in percent<br>3. Raw or change scores in Brain Index
