# Supplementary figures and images for: Effect of brain-computer interface training based on non-invasive electroencephalography using motor imagery on functional recovery after stroke - a systematic review and meta-analysis
Source: BMC Neurol. 2020 Oct 22;20:385. doi: 10.1186/s12883-020-01960-5 (PMC7584076; doi:10.1186/s12883-020-01960-5)

## Slide 1
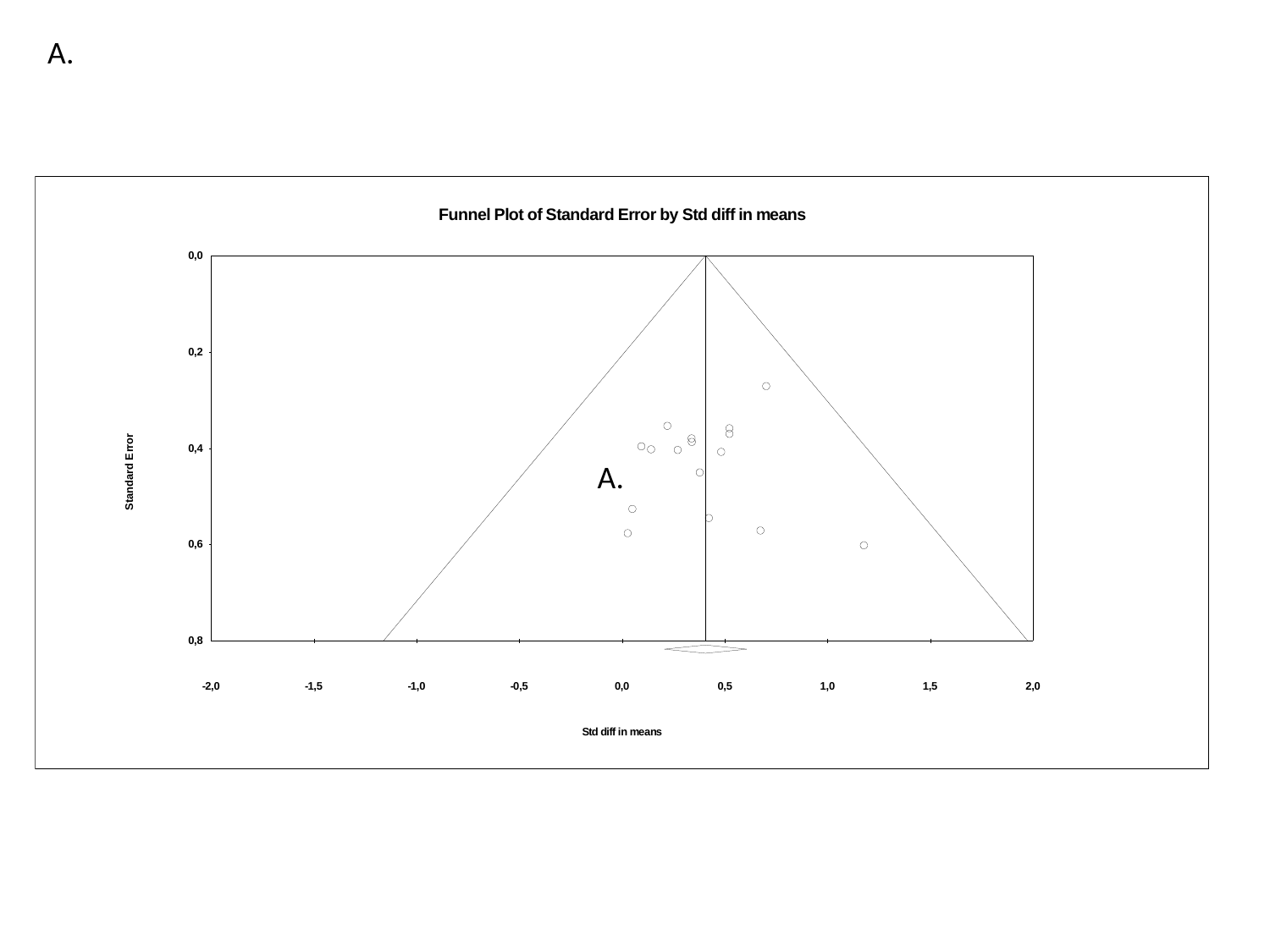

A.
A.

## Slide 2
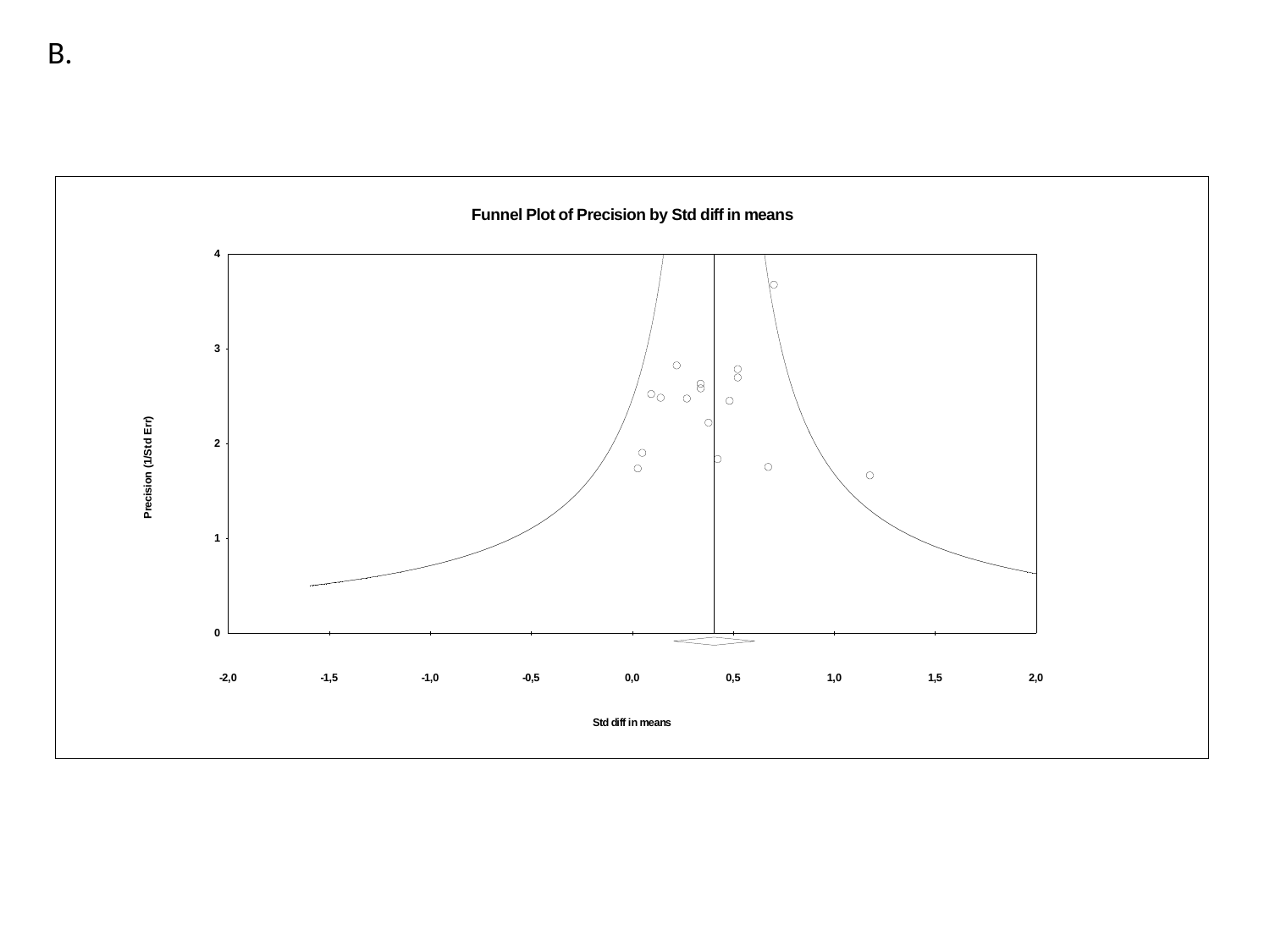

B.

## Slide 3
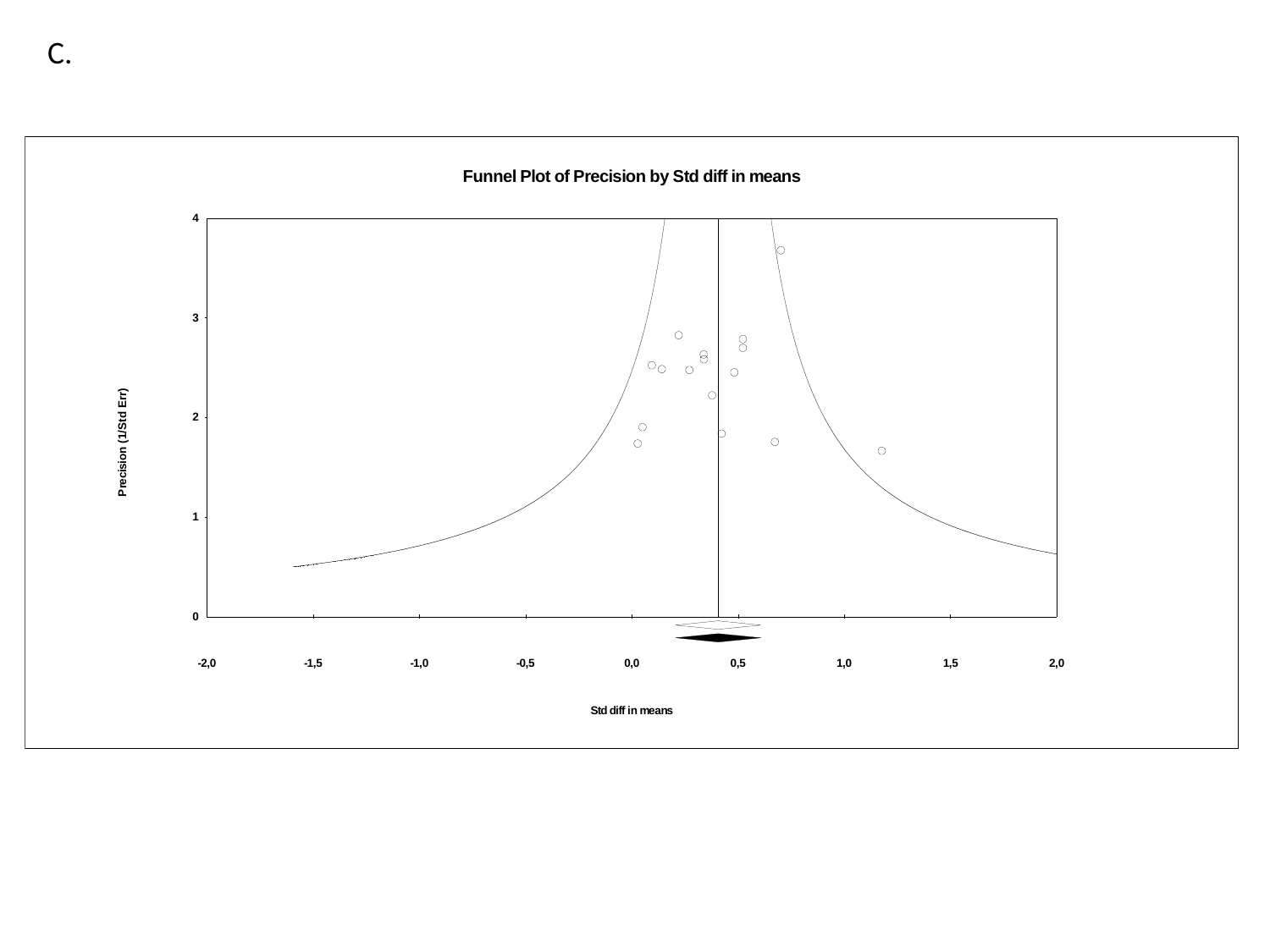

C.

Supplement: Supplementary file 3 — Additional file 3. Figure AM1_Funnel plots. [file 12883_2020_1960_MOESM3_ESM.pptx]

## Slide 1
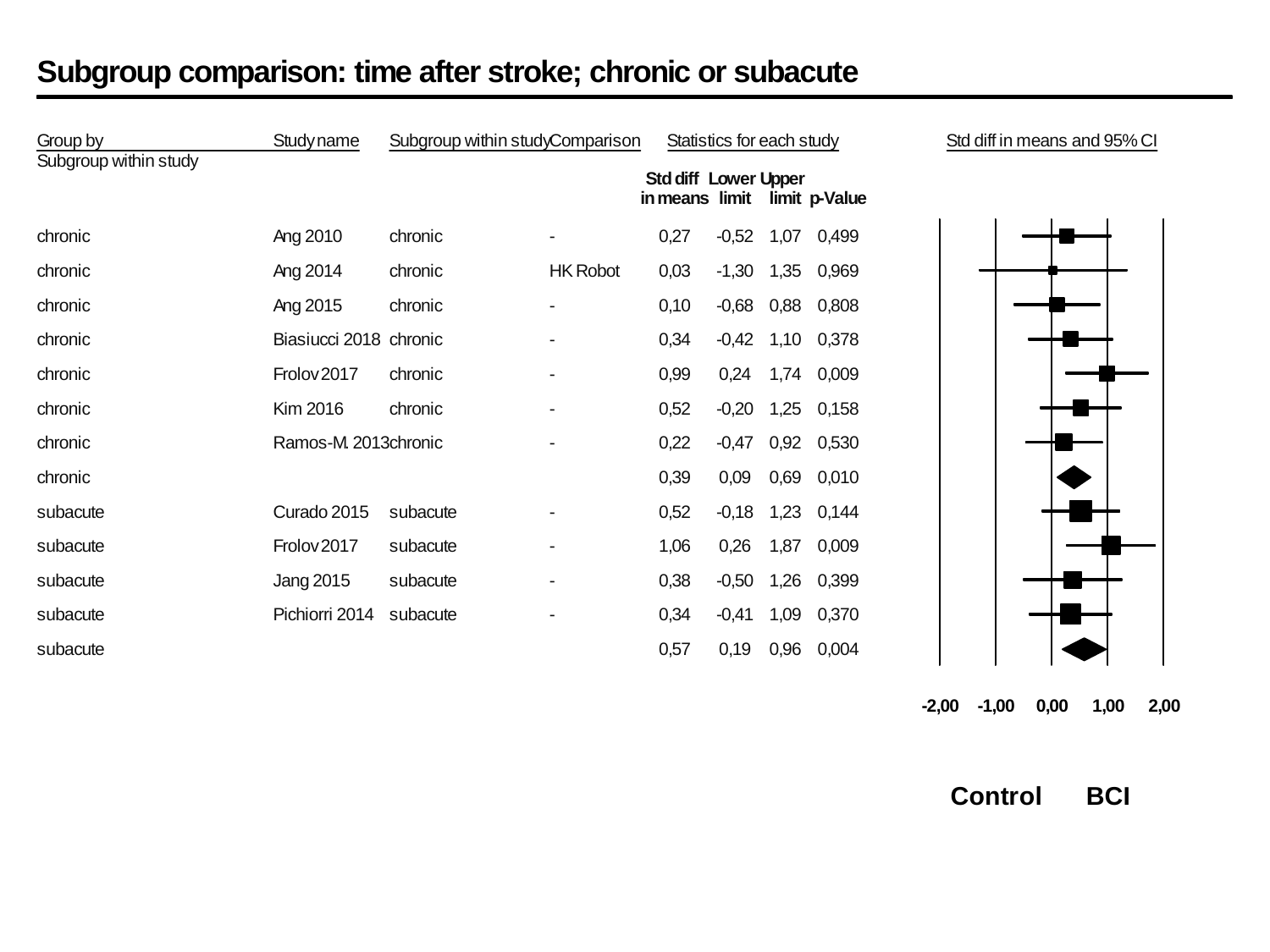

## Slide 2
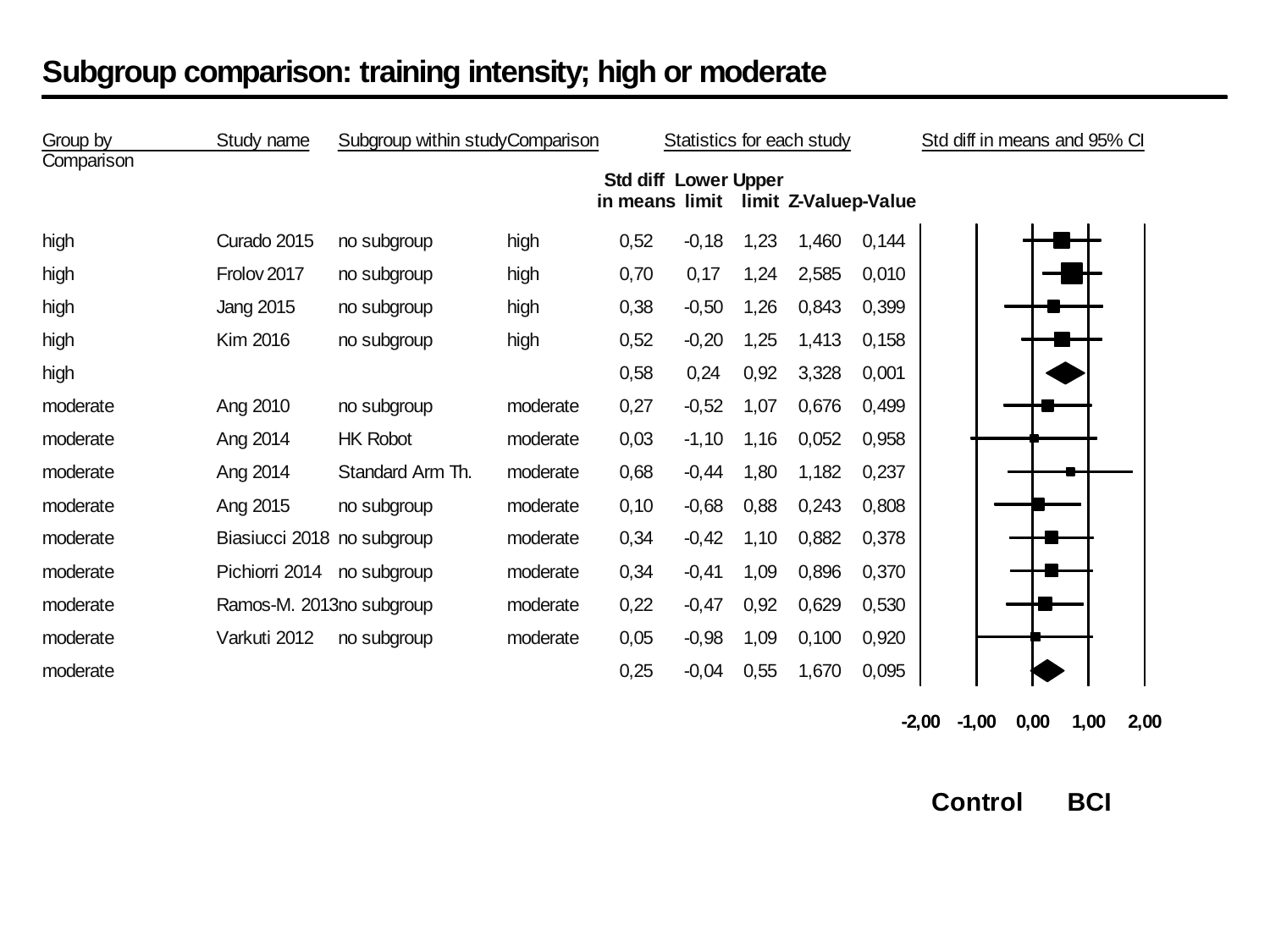

## Slide 3
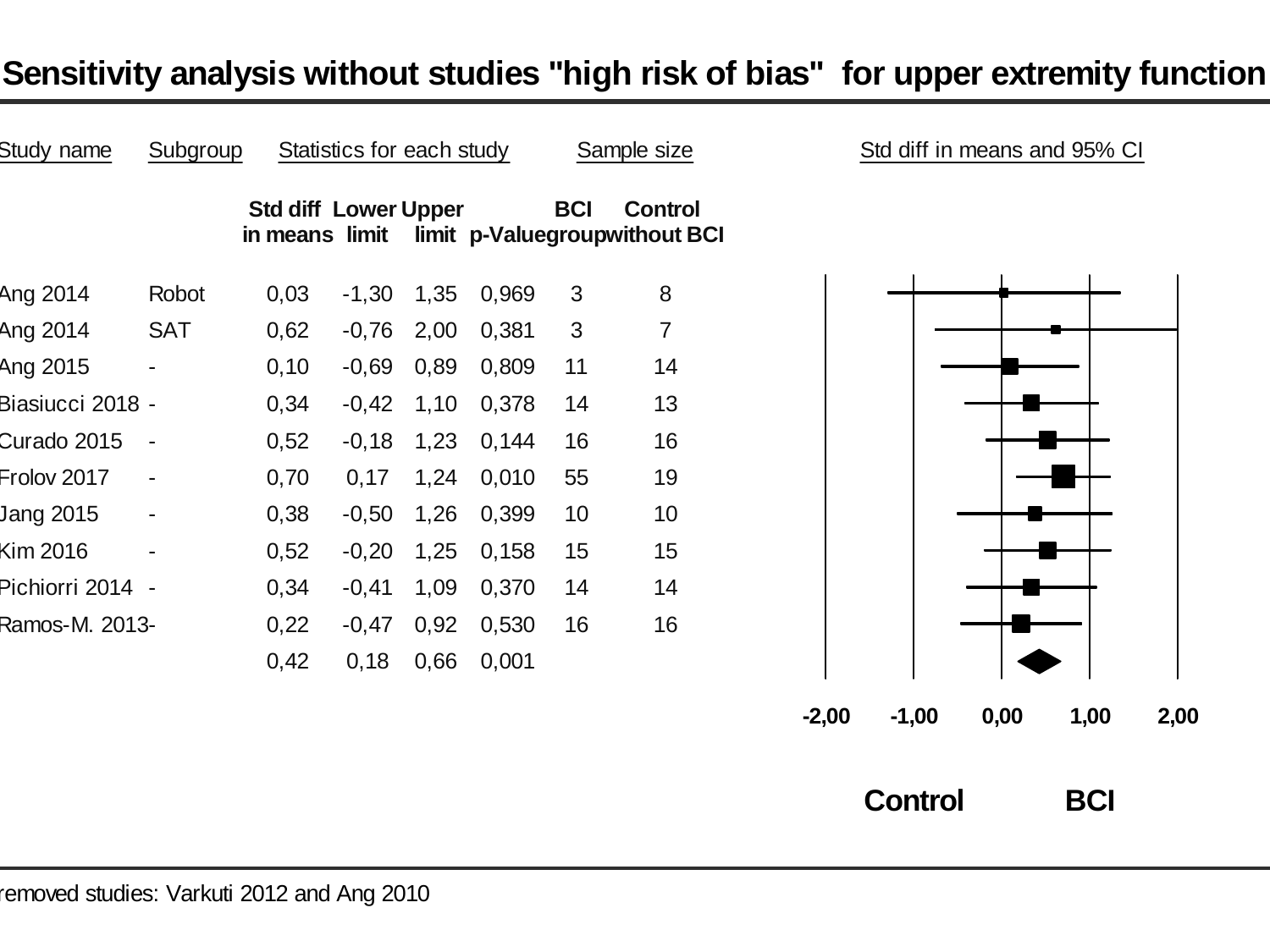

Supplement: Supplementary file 4 — Additional file 4. Figure AM2_Subgroup analyses: Time since stroke, training intensity and sensitivity analysis. [file 12883_2020_1960_MOESM4_ESM.pptx]

## Slide 1
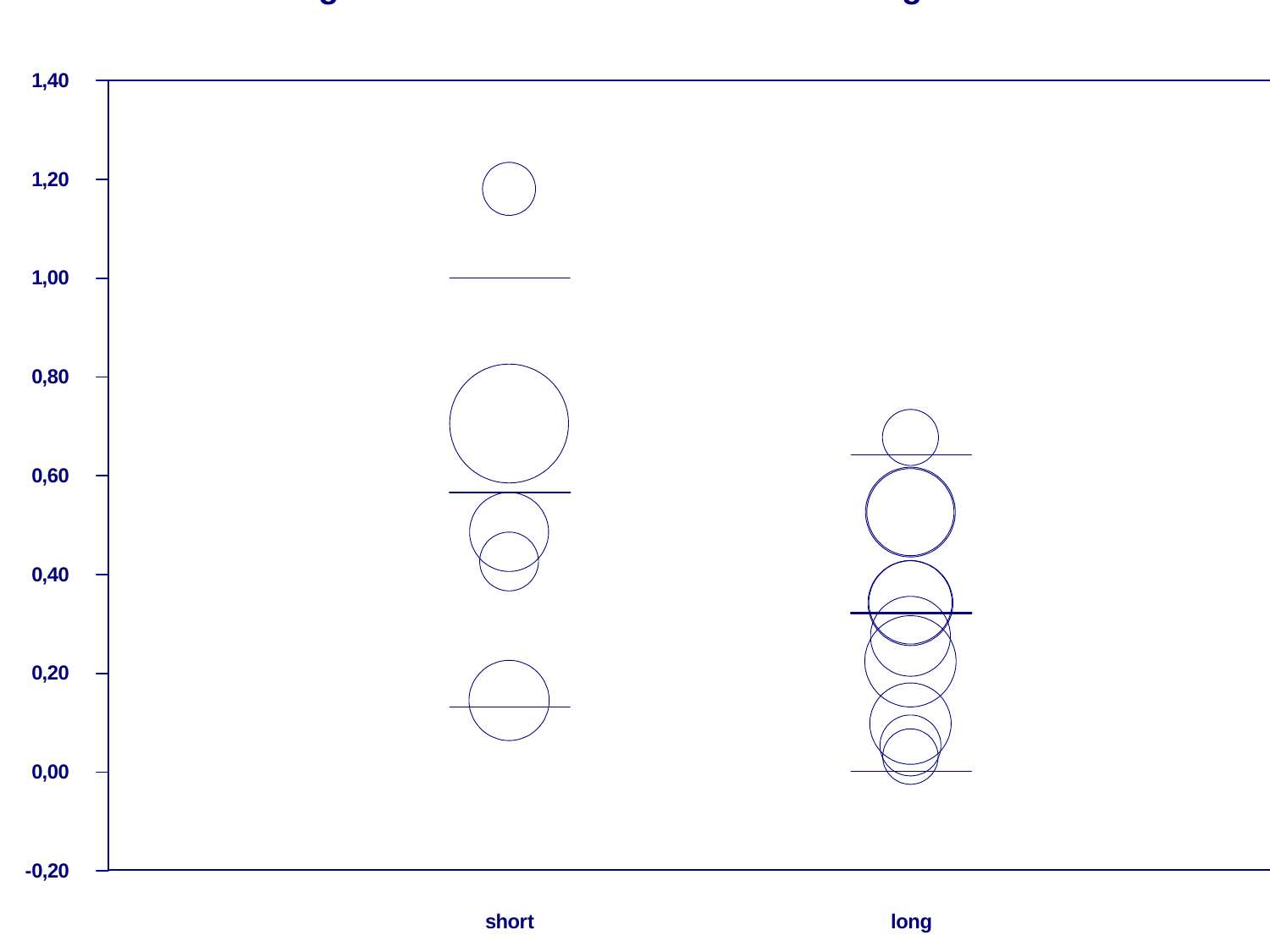

A.

## Slide 2
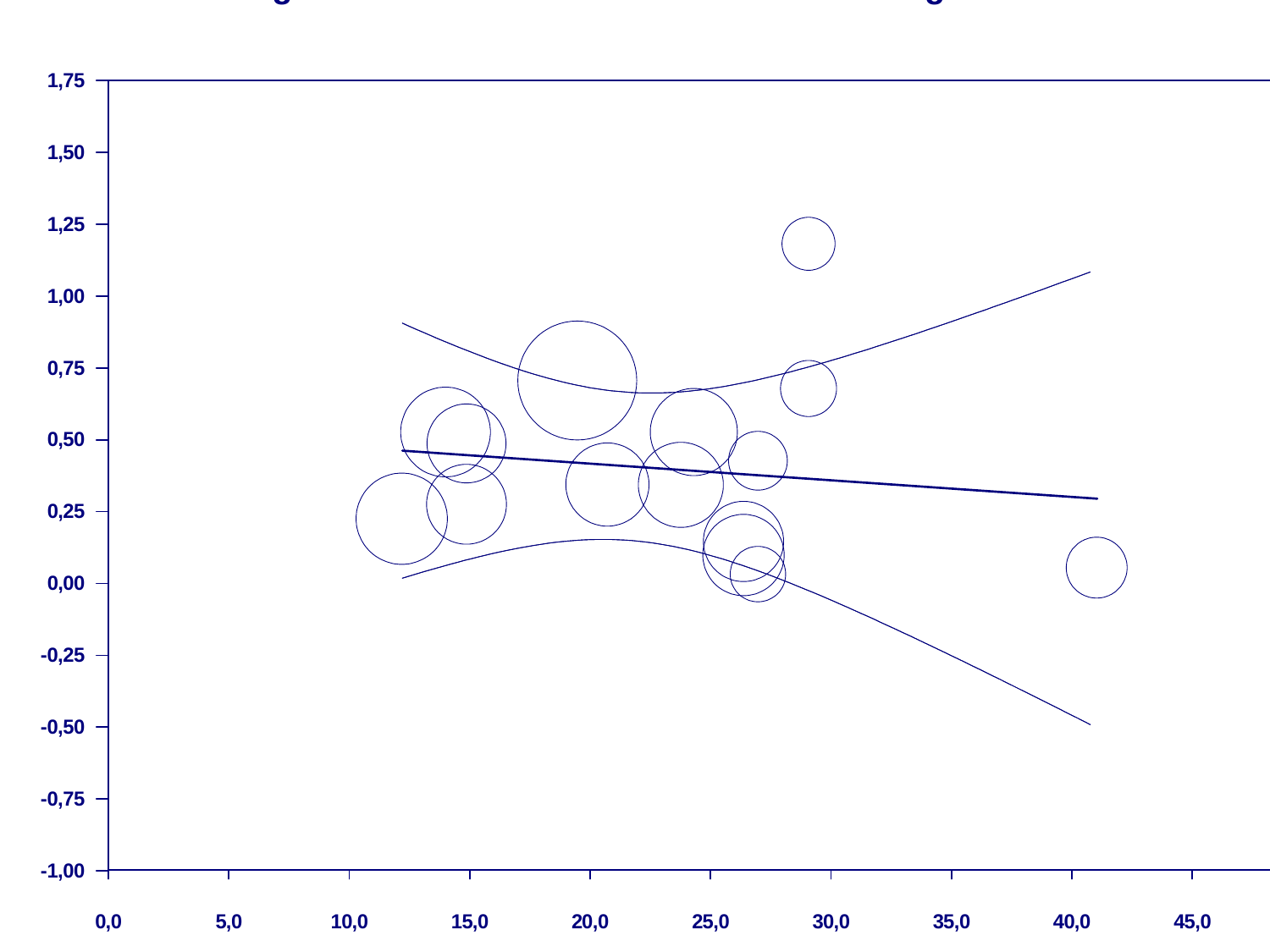

B.

Supplement: Supplementary file 6 — Additional file 6. Figure AM3_Meta Regression [file 12883_2020_1960_MOESM6_ESM.pptx]
