# Supplementary material for: Effect of brain-computer interface training based on non-invasive electroencephalography using motor imagery on functional recovery after stroke - a systematic review and meta-analysis
Source: BMC Neurol. 2020 Oct 22;20:385. doi: 10.1186/s12883-020-01960-5 (PMC7584076; doi:10.1186/s12883-020-01960-5)
Supplement: Supplementary file 5 — Additional file 5. Table AM1_Subgroup analysis: Time duration and FU. [file 12883_2020_1960_MOESM5_ESM.docx]

1. **Training duration analysis**

**Table AM1.** Random effects analysis of subgroup comparison of different time points of the collection of assessments in meta-analysis for BCI of upper extremity

| Subgroups | Point estimate | Confidence interval (95%) | Z-value | p-value | Q-value | df | p-value | I^2^ |
| --- | --- | --- | --- | --- | --- | --- | --- | --- |
| Short duration training | 0.54 | 0.19 to 0.89 | 3.06 | 0.002 | 2.43 | 5 | 0.659 | 0.00 |
| Long duration training | 0.31 | 0.06 to 0.56 | 2.44 | 0.016 | 1.83 | 9 | 0.994 | 0.00 |
| Short-term follow-up | 0.31 | -0.12 to 0.74 | 1.400 | 0.162 | 1.84 | 4 | 0.765 | 0.00 |
| Long-term follow up | 0.56 | 0.01 to 1.11 | 1.99 | 0.047 | 1.80 | 2 | 0.407 | 0.00 |
| **Between effect** | -- | -- | -- | -- | 1.44 | 3 | 0.695 | -- |
